# Structure of full-length TSH receptor in complex with antibody K1-70™

**DOI:** 10.1530/JME-22-0120

**Published:** 2022-09-06

**Authors:** Ricardo Núñez Miguel, Paul Sanders, Lloyd Allen, Michele Evans, Matthew Holly, William Johnson, Andrew Sullivan, Jane Sanders, Jadwiga Furmaniak, Bernard Rees Smith

**Affiliations:** 1 FIRS Laboratories, RSR Ltd, Parc Ty Glas, Llanishen, Cardiff, UK

**Keywords:** TSHR, cryo-EM, autoantibodies, autoimmunity, structure

## Abstract

Determination of the full-length thyroid-stimulating hormone receptor (TSHR) structure by cryo-electron microscopy (cryo-EM) is described. The TSHR complexed with human monoclonal TSHR autoantibody K1-70™ (a powerful inhibitor of TSH action) was detergent solubilised, purified to homogeneity and analysed by cryo-EM. The structure (global resolution 3.3 Å) is a monomer with all three domains visible: leucine-rich domain (LRD), hinge region (HR) and transmembrane domain (TMD). The TSHR extracellular domain (ECD, composed of the LRD and HR) is positioned on top of the TMD extracellular surface. Extensive interactions between the TMD and ECD are observed in the structure, and their analysis provides an explanation of the effects of various TSHR mutations on TSHR constitutive activity and on ligand-induced activation. K1-70™ is seen to be well clear of the lipid bilayer. However, superimposition of M22™ (a human monoclonal TSHR autoantibody which is a powerful stimulator of the TSHR) on the cryo-EM structure shows that it would clash with the bilayer unless the TSHR HR rotates upwards as part of the M22™ binding process. This rotation could have an important role in TSHR stimulation by M22™ and as such provides an explanation as to why K1-70™ blocks the binding of TSH and M22™ without activating the receptor itself.

## Introduction

The G-protein coupled receptor (GPCR) for thyroid-stimulating hormone (TSH) receptor (TSHR) has a key role in the regulation of thyroid function and is a major thyroid autoantigen (Rees Smith *et al.* 1988, Rapoport *et al.* 1998, Morshed *et al.* 2012, Furmaniak *et al.* 2015). In particular, TSHR autoantibodies (TRAbs) bind to the receptor in such a way as to mimic TSH action and cause the hyperthyroidism of Graves’ disease (Rees Smith *et al.* 1988, Rapoport *et al.* 1998, Furmaniak *et al.* 2022). TRAbs also act on TSHRs in the orbit and have an important role in the eye signs of Graves’ disease (Bahn 2010, Furmaniak *et al.* 2022).

TSH and TRAbs bind principally to the concave surface of the receptor’s leucine-rich-repeat domain (LRD, amino acids 22–279). The crystal structures of the human TSHR LRD (amino acids, 22–260; TSHR260) bound to the thyroid-stimulating human monoclonal autoantibody (MAB) M22™ (Sanders *et al.* 2003) and the blocking type MAB K1-70™ (Evans *et al.* 2010, Furmaniak *et al.* 2019) have been solved at 2.55 and 1.9 Å resolution, respectively (Sanders *et al.* 2007, 2011). Also the crystal structure of a thermostable ligand-free TSHR LRD has been solved at 2.83 Å resolution (Miller-Gallacher *et al.* 2019). Also, the crystal structure of the TSHR LRD (Sanders *et al.* 2007) and the crystal structure of the FSHR extracellular domain (ECD) (Jiang *et al.* 2012) allowed structural modelling of the full-length TSHR by different groups (Davies *et al.* 2014, Brüser *et al.* 2016, Schaarschmidt *et al.* 2016, Kleinau *et al.* 2017).

We now describe the structure of the full-length TSHR (amino acids 22–764) in complex with K1-70™ Fab determined by cryo-electron microscopy (cryo-EM).

## Materials and methods

### Preparation of TSHR–K1-70™ complexes

Full-length human TSHR (amino acids 22–764) was expressed in CHO-K1 cells as described previously (Oda *et al.* 1998). The cells were resuspended in 10 mmol/L Tris–HCl pH 7.5 supplemented with complete protease inhibitors (Roche), homogenised and centrifuged at 12,000 ***g*** for 30 min at 4°C to obtain a crude membrane preparation. This was suspended in 10 mmol/L Tris–HCl pH 7.5, 50 mmol/L NaCl, re-sedimented and re-suspended in the same buffer.

K1-70™ IgG was purified from heterohybridoma culture supernatants, Fab was prepared using mercuripapain (Sanders *et al.* 2011) and added to the TSHR membrane preparation. The mixture containing 10 μg/mL of K1-70™ Fab was then incubated for 1 h at 20°C followed by centrifugation at 12,000 ***g*** for 45 min at 4°C and the pellets were homogenised in 10 mmol/L Tris–HCl pH 7.5, 50 mmol/L NaCl, 2% lauryl maltose neopentyl glycol (LMNG), 0.2% cholesteryl hemisuccinate (CHS) containing 25 µg/mL K1-70™ Fab. After incubation for 2 h at 4°C, the homogenate was centrifuged at 90,000 ***g*** for 1 h at 4°C and the supernatant containing solubilised TSHR–K1-70™ Fab complex was stored in aliquots at −70°C.

### Purification of TSHR–K1-70™ complexes

A mouse monoclonal TSHR antibody (14C4) that binds to a conformational epitope within amino acids 22–260 of the TSHR (but distinct from the K1-70™ binding site) was coupled to CNBr-activated Sepharose 4B (Sigma-Aldrich) and a 3 mL column was used to affinity purify solubilised TSHR–K1-70™ Fab complex. After washing with 6 column volumes of 50 mmol/L Tris–HCl pH 8.0,150  mmol/L NaCl, 0.2% LMNG and 0.02% CHS containing 2 µg/mL of K1-70™ Fab, the complex was eluted with 0.1 mmol/L sodium citrate pH 4.5, 0.2% LMNG and 0.02% CHS, 2 µg/mL K1-70™ Fab. One-millilitre fractions were collected and dialysed into 50 mmol/L Tris pH 8.0, 150 mmol/L NaCl, 0.02% LMNG and 0.002% CHS.

**Figure 1:**
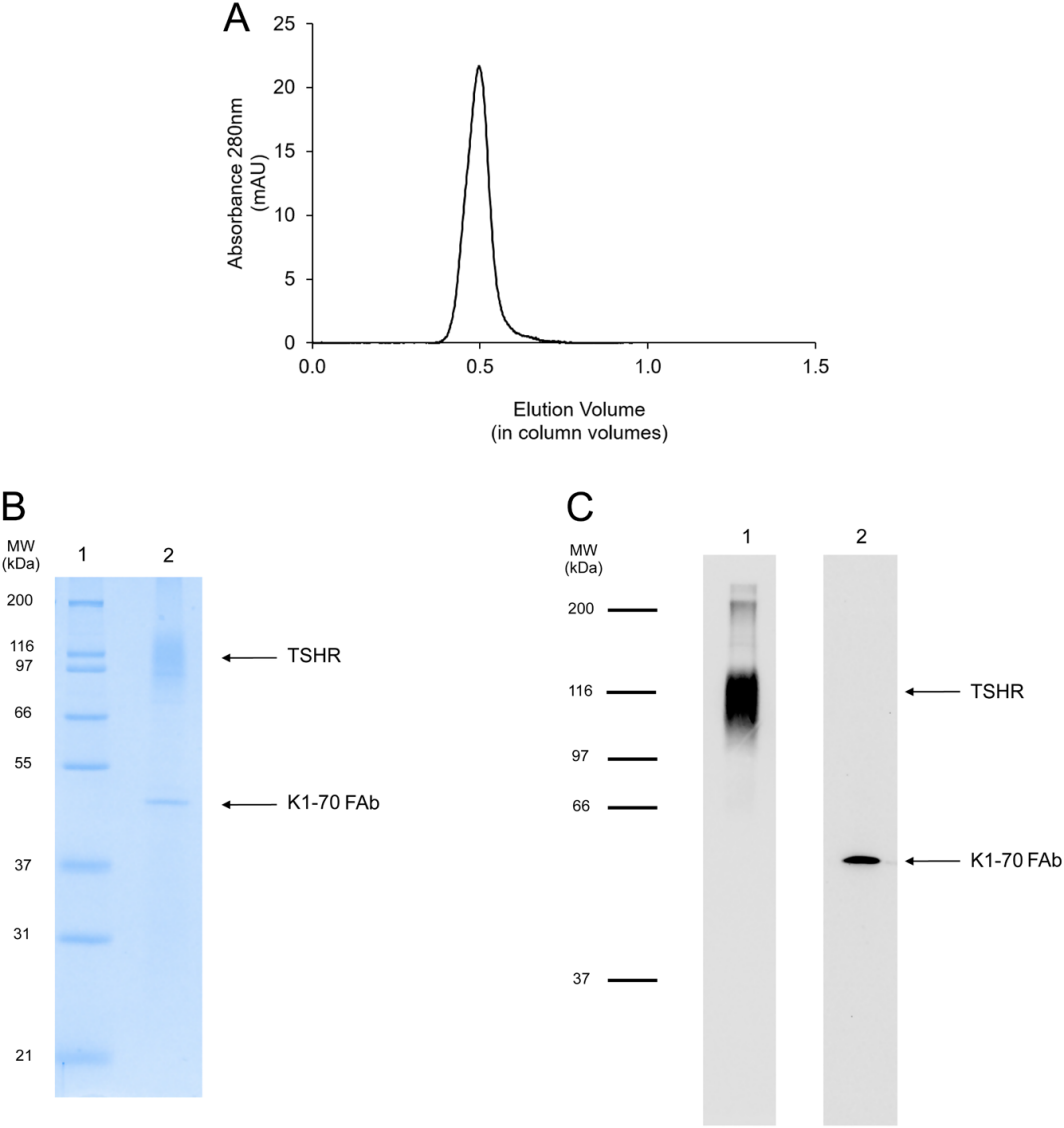
(A) Analysis of purified TSHR–K1-70™ complex by size exclusion chromatography (Superdex 200 XK 16-100 column run in 50 mmol/L Tris–HCl pH 8.0, 150 mmol/L NaCl, 0.02% LMNG and 0.002% CHS). (B) Analysis of purified TSHR–K1-70™ complex by SDS-PAGE (12% acrylamide gel) under non-reducing conditions. The positions of the molecular weight standards are shown and the positions of the TSHR and the K1-70™ Fab are marked (lane 1: molecular weight standards; lane 2: purified TSHR–K1-70™ complex). (C) Western blotting analysis of purified TSHR–K1-70™ complex. Lane 1: blotting with mouse monoclonal TSHR antibody 8E2 labelled with horseradish peroxidase; lane 2: blotting with mouse anti-human IgG (Fab-specific) MAB labelled with horseradish peroxidase. See text for experimental details.

After dialysis, the complex was concentrated using a 10 kDa MWCO spin concentrator (Thermo Scientific) and run on a Superdex 200 XK 16-100 column (GE Healthcare) in 50 mmol/L Tris–HCl pH 8.0, 150 mmol/L NaCl, 0.02% LMNG and 0.002% CHS. The fractions containing the complex (Fig. 1) were collected and concentrated to 1 mg/mL for cryo-EM.

### Analysis of TSHR–K1-70™ complexes

During purification of the complex, total protein concentration was determined by Bradford assay (Biorad). Also, the presence of K1-70™ Fab and the presence of the TSHR in various fractions were monitored. Briefly, 150 μL of TSHR–K1-70™ complex was incubated overnight at 4°C in ELISA plate wells coated with a mouse MAB which binds to the C-terminus of the TSHR. The wells were washed thrice with wash buffer (50 mmol/L NaCl, 10 mmol/L Tris–HCl pH 7.8, 0.05% LMNG) and 100 μL of either TSHR MAb 14C4 labelled with horse radish peroxidase (14C4-POD; RSR Ltd, Cardiff, UK) or anti-human Fab-specific antibody labelled with horse radish peroxidase (Sigma Aldrich) was added and the samples were incubated for 20 min at room temperature. The plate wells were then washed twice with wash buffer, once with water before the addition of 100 μL 3,3’,5,5’ tetramethylbenzidine substrate and a further 35 min incubation in the dark at room temperature. The reaction was then stopped by the addition of 50 μL of 0.5M H_2_SO_4_ and the absorbance was read at 450 nm using an ELISA plate reader.

The purity of the final complex was assessed by 12% non-reduced sodium dodecyl sulphate-PAGE (SDS-PAGE) (Laemmli 1970) and Western blotting (Birk & Koepsell 1987) with anti-human Fab-specific antibody labelled with peroxidase (Sigma Aldrich) to detect the presence of K1-70™ Fab and peroxidase-labelled mouse MAB to the N-terminus of the TSHR (8E2; Oda *et al.* 2000) to detect TSHR. The concentration of the purified complex was determined by optical density measurements at 280 nm using the extinction coefficient of 1.330 mL/mg/cm calculated from the amino acid sequence using ProtParam (https://www.expasy.org/resources/protparam).

### Cryo-EM grid preparation and data collection

The purified complex (3 μL of 1 mg/mL) was applied to UltrAufoil R1.2/1.3 300 mesh grids and the grids glow discharged once per slide, the foil side being the most recently charged. A total of 12 cryo-TEM grids were prepared using Vitrobot Mark IV (Life Sciences). The data were collected from the UltrAufoil grid from undiluted complex (1 mg/mL) with 0 blotting force and 3 s blot time.

Data collection was performed on a Titan Krios 300kV (at Cryo-EM Facility, Department of Biochemistry, University of Cambridge, UK) using 130,000× magnification, corresponding to a pixel size of 0.652 Å/pixel. The data were collected with a Falcon 3 Direct Detector in electron counting mode. A total dose of 47.3 e/Å^2^ and 50 fractions were used for recording movies during each exposure which lasted 501.34 s and a defocus range from −2.4 to −1.0 µm with a defocus step of 0.2 µm.

A total of 8219 movies were collected during the data collection session. WARP (Tegunov & Cramer 2019) identified about 1,600,000 particles which were then passed into Cryo-SPARC processing for 3D reconstruction (Punjani *et al.* 2017).

### Molecular replacement and refinement

The electron density of the TSHR–K1-70™ complex showed the TSHR bound to K1-70™ Fab. The crystal structure of the TSHR LRD in complex with K1-70™ Fab (Sanders *et al.* 2011; PDB-Id: 2xwt) and the model of TSHR (AlphaFold database: https://alphafold.ebi.ac.uk/; Tunyasuvunakool *et al.* 2021) were fitted into the electron density.

The structure was manually refined using COOT v0.9 (Emsley *et al.* 2010) as the first step. Then, the Refinement Cascade protocol within the Discovery Studio 2021 suite of programs (BIOVIA Dassault Systèmes 2021) was run to automatically refine the structure of the complex. Finally, minimisation was carried out with MODELLER (Sali & Blundell 1993, Webb & Sali 2016) followed by an automatic All-atom Refine protocol with COOT. This process was repeated three times.

## Results

### TSHR–K1-70™ complex

The peak fractions from the preparative size exclusion chromatography (SEC) of the TSHR–K1-70™ complex were pooled and concentrated to 1 mg/mL. This preparation bound to 14C4-POD and to anti-Fab-POD, confirming the presence of both TSHR and K1-70™ Fab components in the complex and analytical SEC of the preparation, indicated purity of >95% (Fig. 1A). On non-reduced SDS-PAGE, the complex separated into two components corresponding to TSHR (molecular weight 89–116 kDa) and K1-70™ Fab (47 kDa) (Fig. 1B). Western blotting analysis also confirmed the presence of both the TSHR and K1-70™ Fab in the complex (Fig. 1C).

### Full-length human TSHR structure

**Figure 2:**
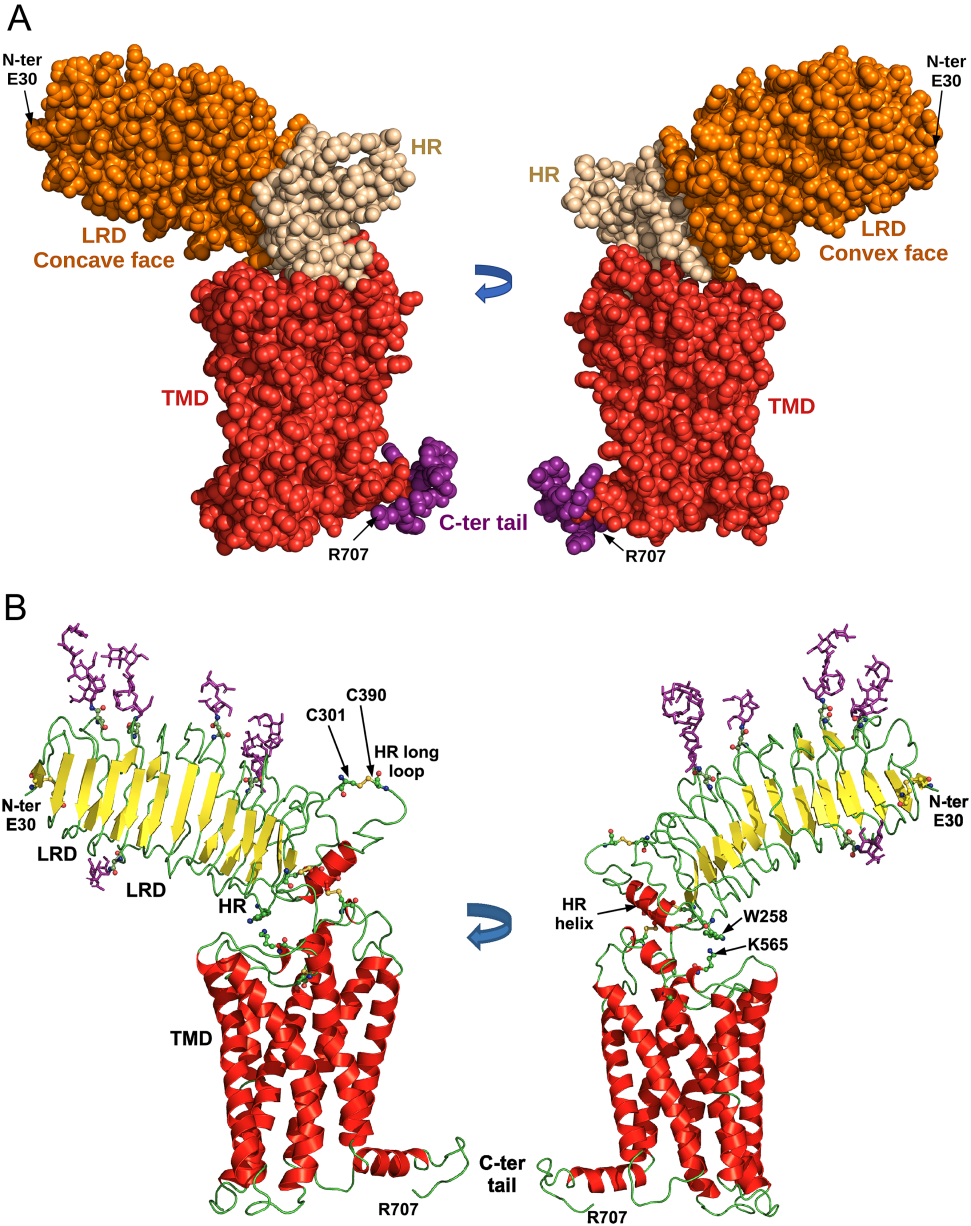
Structural features of the full-length TSHR. (A) TSHR structure (amino acids E30 to R707) in two views rotated 180^o^. The structure is a space fill representation with the leucine-rich-repeat domain (LRD) in orange, the hinge region (HR) in beige and the transmembrane domain (TMD) in red with the intracellular C-terminus in purple. (B) TSHR structure in two views rotated 180^o^. The structure is in cartoon representation with the disulphide bonds and glycosylation sites in ball and stick representation with oxygen in red, nitrogen in blue and sulphur in yellow. The glycans are shown in purple. The LRD, HR and TMD are marked. Cys301 forms a disulphide bond with Cys390 and the residues in between (amino acids 302–389) are missing from the structure.

Processing of the cryo-EM data resulted in a final structure for the TSHR–K1-70™ Fab complex at 3.32 Å overall resolution (Supplementary Fig. 1 and Supplementary Table 1, see section on supplementary materials given at the end of this article). It consists of the TSHR bound to K1-70™ Fab with the electron density showing the TSHR structure from Glu30 to Arg707. This is composed of three domains, the ECD (amino acids 22–409) consisting of the LRD (amino acids 22–279) and hinge region (HR; amino acids 280–409), the TMD and the intracellular C-terminus (amino acids 410–764) (Fig. 2A and B). The structure of the ECD, formed by the LRD and HR, is that of a typical leucine-rich-repeat (LRR) structure with 11 repeats in the LRD and 1 repeat in the HR. Each LRR consists of a parallel β-strand on its concave surface while the N-terminal cap (N-cap) has an additional β-strand antiparallel to the β-strand of the first repeat (Supplementary Fig. 2A). The LRD and the HR form a continuum structure with the N-cap, with two disulphide bonds, and a C-terminal cap (C-cap) with three disulphide bonds and an alpha helix. One of the two N-terminal disulphide bonds between Cys31 and Cys41 is visible in the structure while the other one between Cys24 and Cys29 is not. All three intra-domain disulphide bonds which form the C-cap are visible in the structure between Cys283 and Cys398, Cys284 and Cys408, and Cys301 and Cys390. However, TSHR residues from Asn302 to Ile389, which form part of the unstructured long hinge loop, are not visible in the structure.

The cryo-EM structure shows five N-linked glycans in the LRD in all the positions expected from the sequence consensus for N-linked glycosylation and shown in the TSHR LRD crystal structures (amino acids Asn77, Asn99, Asn113, Asn177 and Asn198). A further glycosylation site, Asn302, is not visible in the cryo-EM structure (Fig. 2A, B and Supplementary Fig. 2A).

The structure of the TSHR TMD (Supplementary Fig. 2B) is that of a typical GPCR 7 helices TMD (Núñez Miguel *et al.* 2017*a*) with an 8th helix (TM8) parallel to the membrane. The long extracellular loop (ECL) 2 is disulphide bonded to transmembrane helix (TM) 3 through cysteine residues 569 and 494. The long ECL1 loop has an α-helix close to its C-terminus and proline kinks are seen in the structure of helices TM6 and TM7 which correspond to a change in direction of the helix axis. Helix TM4 is broken by the Gly–Gly sequence at residues 544 and 545 and a proline distortion is seen at the extracellular end of the helix.

Cation–π interactions are observed between LRD residue Trp258 (10th LRR) and TMD residue Lys565 of ECL2. Residues Lys261 and Tyr279, in the 10th and 11th LRR, respectively, and Ser281, Cys284 and Asn288 in the HR helix interact with residues in ECL1 (Table 1). Furthermore, residue E404 in the C-cap interacts with both ECL2 and the extracellular part of TMD helix 7.

**Table 1:** Interactions between the TSHR ECD and TMD.

TSHR ECD		TSHR TMD		Interaction types
Residue	Location	Residue	Location	
W258	10th Repeat	K565	ECL2	Cation–π
K261	10th Repeat	I486	ECL1	Induced dipole
		T490	ECL1	Hydrogen bond, hydrophobic
Y279	11th Repeat	I486	ECL1	Hydrophobic
		T490	ECL1	Polar
S281	HR-helix	Y482	ECL1	Hydrogen bond, induced dipole
		A485	ECL1	Hydrophobic
		I486	ECL1	Induced dipole
C284	HR-helix	Y482	ECL1	Hydrophobic
N288	HR-helix	Y482	ECL1	Hydrogen bond, hydrophobic, induced dipole
E404	C-ter cap	V566	ECL2	Hydrophobic, induced dipole
		S567	ECL2	Induced dipole
		I568	ECL2	Induced dipole
		K660	TM7	Hydrophobic
		V656	TM7	Induced dipole
F405	P10 seg	Q489	ECL1	Hydrophobic, induced dipole
		A564	ECL2	Induced dipole
		K565	ECL2	Hydrophobic, induced dipole
		V566	ECL2	Hydrophobic
		S567	ECL2	Polar, hydrophobic, induced dipole
P407	P10 seg	Y481	ECL1	Hydrophobic
C408	P10 seg	Y481	ECL1	Hydrogen bond, induced dipole
		Y482	ECL1	Hydrogen bond, induced dipole
E409	P10 seg	T477	ECL1	Induced dipole
		S479	ECL1	Polar, hydrophobic, induced dipole
		E480	ECL1	Hydrogen bond, induced dipole
		Y481	ECL1	Hydrogen bond, hydrophobic, induced dipole
D410	P10 seg	R418	TM1	Salt bridge (2), ion pair (3), induced dipole, long-range charge–charge
		D474	TM2	Polar, induced dipole
		T477	ECL1	Polar, induced dipole
		S479	ECL1	Induced dipole
I411	P10 seg	V473	TM2	Hydrophobic
		D474	TM2	Polar, hydrophobic, induced dipole
		Y481	ECL1	Hydrophobic
		S567	ECL2	Induced dipole
M412	P10 seg	R418	TM1	Polar, hydrophobic, induced dipole
		I568	ECL2	Hydrophobic
		K660	TM7	Polar, induced dipole
		V664	TM7	Induced dipole
G413	P10 seg	K415	N-ter	Polar, induced dipole
Y414	P10 seg	F416	TM1	Hydrogen bond, aromatic, hydrophobic, induced dipole
		L417	TM1	Polar, hydrophobic, induced dipole
		S657	TM7	Hydrogen bond
		I661	TM7	Hydrophobic

ECLX, extracellular loop X; HR-helix, hinge region helix; P10 seg, P10 segment; TMX, transmembrane helix X.

The TSHR P10 peptide (Brüser *et al.* 2016) (amino acids 405–414 of the TSHR) is visible in the cryo-EM structure and forms the C-terminal half of the linker between the TSHR ECD and the TMD. The P10 peptide interacts with TMD residues in ECL1, ECL2, TM1, TM2 and TM7 (Table 1) forming two salt bridges, six hydrogen bonds, three ion pairs and several polar, aromatic, hydrophobic and induced dipole interactions. The intracellular C-terminal tail of the TSHR is visible up to amino acid 707 with the last 57 amino acids not visible in the structure.

A structural superimposition of the TSHR ECD residues 30–257, which are present in all four available TSHR structures (cryo-EM TSHR–K1-70™, TSHR260–K1-70™ (PDB code 2XWT), TSHR260–M22™ (PDB code 3G04) and the thermostable TSHR260–JMG55™ (Miller-Gallacher *et al.* 2019), gave low root-mean-square deviation (RMSD) values (0.51–0.75 Å; Supplementary Table 2). A superimposition of the hinge region (residues 261–301 and 390–411) of our cryo-EM structure of the TSHR with the hinge region of the AlphaFold TSHR model (Tunyasuvunakool *et al.* 2021) gave a high RMSD of 3.5 Å mainly due to a longer hinge helix in the AlphaFold models of all 3 glycoprotein hormone receptors (GPHRs).

### K1-70™ structure

The K1-70™ Fab structure consists of K1-70™ heavy chain (HC) residues Gln1 to Ser229 and light chain (LC) residues Ser2 to Ala212 and is the structure of a typical Fab fragment.

### TSHR–K1-70™ complex structure

**Figure 3:**
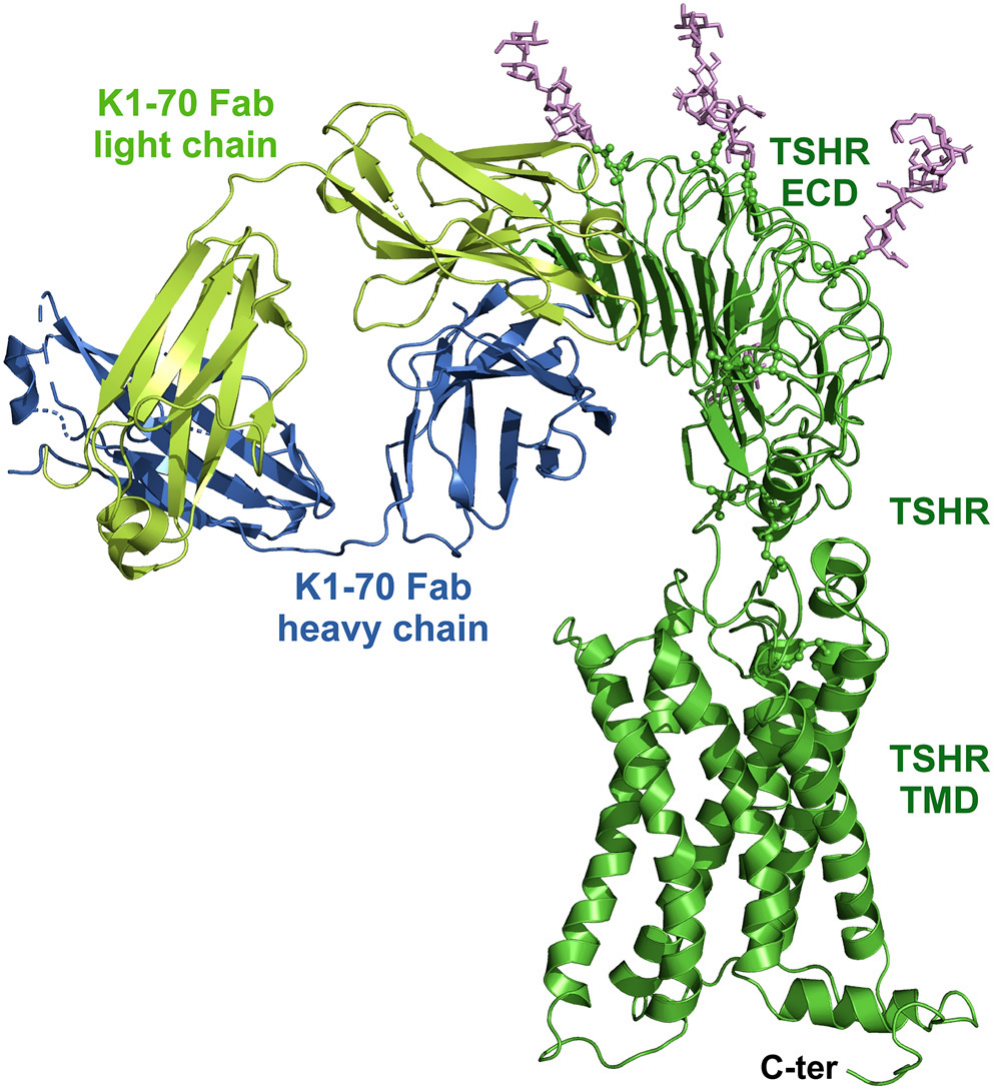
Structural features of the full-length TSHR in complex with the blocking monoclonal autoantibody K1-70™. The TSHR–K1-70™ Fab structure is in cartoon representation with the glycans shown in purple sticks. The K1-70™ heavy chain is shown in blue, the K1-70™ light chain in light green and the TSHR in green. The TSHR, extracellular domain (ECD), transmembrane domain (TMD) and intracellular C-terminus are marked.

K1-70™ Fab binding is principally to the concave surface of the TSHR LRD (Fig. 3 and Supplementary Fig. 4), which has no glycans attached. K1-70™ Fab HC and LC bind to the N-terminal repeats of the TSHR (Tables 2 and 3) from amino acid Glu35 in the TSHR N-cap to Lys183 in the 7th LRR. There is a mixture of an extensive hydrogen bonding and salt bridge network (20 hydrogen bonds and salt bridges), 5 ion pairs, 13 polar interactions and 13 hydrophobic/aromatic contacts. Differences in some of the interactions seen in the cryo-EM structure of the full-length TSHR bound to K1-70™ Fab compared to the crystal structure of the TSHR LRD in complex with K1-70™ Fab are most likely due to side chain flexibility in the interacting amino acids. There are no interactions between the K1-70™ Fab and either the HR or the TMD.

**Table 2:** Interactions between the TSHR LRD and K1-70 heavy chain.

TSHR LRD		K1-70 Heavy chain residue	Interaction types
Residue	Location		
D36	First repeat	R101	Salt bridge, induced dipole, long-range charge–charge
R38	First repeat	D96	Salt bridge, ion pair (2), induced dipole, long-range charge–charge
		R101	Polar, induced dipole
		Y102	Hydrogen bond, polar, cation–π, induced dipole
K42	First repeat	D31	Hydrogen bond, salt bridge (2), induced dipole
K58	Second repeat	D31	Hydrogen bond, induced dipole
		N32	Induced dipole
		D96	Salt bridge, Hydrogen bond, induced dipole, long-range charge–charge
		W97	Cation–π
I60	Second repeat	D31	Hydrophobic, induced dipole
R80	Third repeat	Y99	Hydrogen bond, induced dipole
		N100	Hydrogen bond, polar, hydrophobic, induced dipole
Y82	Third repeat	W97	Aromatic, induced dipole
		N100	Hydrogen bond, polar, induced dipole
S84	Third repeat	Y52	Polar, induced dipole
		W97	Hydrophobic
H105	Fourth repeat	N98	Polar, induced dipole
E107	Fourth repeat	R58	Long-range charge–charge
		W97	Hydrogen bond, induced dipole
R109	Fourth repeat	W33	Cation–π
		D54	Salt bridge (2), induced dipole, long-range charge–charge
		D56	Salt bridge, ion pair (2), induced dipole, long-range charge–charge
		R58	Polar
		W97	Hydrophobic
N110	Fourth repeat	D54	Hydrogen bond, polar, hydrophobic, induced dipole
R112	Fourth repeat	D54	Long-range charge–charge
K129	Fifth repeat	Y99	Cation–π
F130	Fifth repeat	Y99	Aromatic
F134	Fifth repeat	R58	Cation–π
N135	Fifth repeat	D54	Polar, induced dipole
E157	Sixth repeat	R58	Salt bridge, ion pair, long-range charge–charge

**Table 3:** Interactions between the TSHR LRD and K1-70 light chain.

TSHR LRD		K1-70 Light chain residue	Interaction types
Residue	Location		
E35	N-ter cap	S56	Hydrogen bond, hydrophobic, induced dipole
D36	First repeat	Y49	Polar, hydrophobic, induced dipole
		P55	Polar, induced dipole
		S56	Polar, induced dipole
R80	Third repeat	Y32	Cation–π
		D50	Long-range charge–charge
K102	Fourth repeat	Y32	Hydrogen bond
		D50	Long-range charge–charge
T104	Fourth repeat	Y32	Induced dipole
K129	Fifth repeat	N31	Induced dipole
		S93	Induced dipole
I152	Sixth repeat	S27A	Induced dipole
		G29	Hydrophobic
		S30	Induced dipole
F153	Sixth repeat	S27A	Hydrophobic, induced dipole
		S93	Hydrophobic, induced dipole
		R94	Cation–π, hydrophobic, induced dipole
I155	Sixth repeat	S93	Induced dipole
K183	Seventh repeat	S93	Polar

## Discussion

The TSHR LRD structure seen by cryo-EM is in good agreement with the three LRD structures determined previously by X-ray crystallography (Sanders *et al.* 2007, 2011, Miller-Gallacher *et al.* 2019). All four structures show the extensive concave surface of the LRD forming the binding site for K1-70™ Fab and M22™ Fab. Also, no glycans are seen on the concave surface of the LRD in any of the four structures and this would be expected from the assessment of the consensus sequence for N-linked glycosylation (Asn-Xxx-Ser/Thr, where Xxx is any amino acid except proline; Núñez Miguel *et al.* 2017*b*). The full-length TSHR used to determine the cryo-EM structure was not deglycosylated in the process (in contrast to the deglycosylation carried out during preparation of material for X-ray crystallography), and this provides further evidence of the absence of glycans on the concave surface of the TSHR LRD.

The cryo-EM structure of the TSHR has intact disulphide bonds at five of the six expected disulphide bond sites, confirming the disulphide bond arrangements at the N-terminus, the intra-domain disulphide bonding which forms the C-cap of the HR and the disulphide bond between the ECL2 and TM3 in the TMD. The disulphide bond between Cys301 and Cys390 in the HR long loop is clearly visible in the structure; however, residues 302–389 between these cysteine residues lack clear electron density in this region. This could be due to both flexibility of the long unstructured loop and cleavage of the TSHR into two subunits (A and B; Rees Smith *et al.* 1988), with the removal of approximately 50 amino acids (Chazenbalk *et al.* 1997, Oda *et al.* 2000). Part of the cleaved region (amino acids 316–366) which is not visible in our structure has been reported to bind to neutral TSHR antibodies (Morshed *et al.* 2012).

There are clear interactions between some TSHR ECD and TMD residues. Amino acid Trp258 in the 10th LRR interacts with Lys565 in ECL2 and Lys565 also interacts with F405 (P10 segment). Mutation of the amino acid tryptophan 258 of the TSHR ECD to alanine results in a decrease in the constitutive activity of the TSHR by approximately 50% (unpublished data) and decreased stimulating activity by TSHR stimulating human MABs such as M22™ and K1-18™ (Núñez Miguel *et al.* 2012). Furthermore, TSHR Trp258 is seen to form important interactions with M22™ in the crystal structure of the TSHR-M22™ complex (Sanders *et al.* 2007). Also, mutation of TSHR Lysine 565 to alanine has been reported to stop the constitutive activity of the TSHR and strongly impair hormone-induced signalling activity (Kleinau *et al.* 2007). The decrease in the constitutive activity of the TSHR when the interaction between Trp258 and Lys565 is disrupted by the introduction of alanine at either position 258 or 565 suggests an important role for this interaction in the constitutive activity of the TSHR. No interactions between K1-70™ Fab and TSHR Trp258 are seen in the cryo-EM or crystal structures (Sanders *et al.* 2011). This is consistent with the observation that K1-70™ has no effect on the constitutive activity of the TSHR despite being a powerful inhibitor of TSHR stimulation by TSH and by TRAbs. Furthermore, the luteinizing hormone (LH)/chorionic gonadotrophin (CGR) has no constitutive activity (Van Sande *et al.* 1995) and no interaction is seen between the equivalent residues, Tyr254 and Lys510, in the inactive LH/CGR cryo-EM structure (WT LH/CGR bound to the small molecule antagonist, compound 26) (Duan *et al.* 2021).

Mutations in Ser281 to alanine, asparagine, threonine or isoleucine are well documented for their ability to increase the constitutive activity of the TSHR. In the TSHR cryo-EM structure, Ser281 is involved in interactions with three residues of ECL1 including one hydrogen bond (Table 1). In addition, Ser281 also interacts with residues Cys283, Cys284 and Ala285 of the HR and Asn606 of the P10 segment. Disruption of some or all of these interactions leads to increased constitutive activity of the TSHR.

Amino acid substitutions in TSHR ECL1, ECL2 and ICL3 have previously been shown to increase the constitutive activity of the TSHR, with Ile486 (ECL1) and Ile568 (ECL2) showing strong activating activity (Parma *et al.* 1995). In our cryo-EM structure residues Lys261, Tyr297 and Ser281 in the 10th LRR, 11th LRR and HR helix all interact with Ile486 (ECL1). Furthermore, Glu404 (C-cap of HR) and Met412 (P10 region) interact with Ile568 (Table 1). Disruption of these interactions with amino acid mutations will weaken the binding of the ECD to the TMD and cause the observed increase in constitutive activity.

In the cryo-EM structure, the positioning of the TSHR HR on top of the TMD can be seen for the first time. The P10 region (amino acids 405–414) is visible in the structure and forms the C-terminal half of the linker between the TSHR ECD and the TMD. There are multiple interactions between nine of the ten amino acids in the P10 region with residues in ECL1 and ECL2 of the TMD as well as interactions with TM1, TM2 and TM7 (Table 1). Furthermore, the P10 region residue Cys408 is disulphide bonded to Cys284 of the HR. The presence of this network of interactions with the P10 region would be expected in the inactive conformation of the receptor. The P10 region is the most highly conserved among glycoprotein hormone receptors (Duan *et al.* 2021) and is known to undergo a conformational rearrangement during activation of some class B GPCRs (Zhang *et al.* 2017, 2018). It has been proposed that activation of the TSHR induces a conformational change which leads to the P10 region forming an intramolecular agonist that interacts with the TMD of the receptor (Brüser *et al.* 2016, Krause *et al.* 2020). These proposed conformational changes in the TMD would allow G proteins to bind to the intracellular side of the TSHR. The functionally important ionic lock (salt bridge between Arg519 and Asp619) which stabilises the inactive state of GPCRs (Katritch *et al.* 2012) is also present in the TSHR–K1-70™ structure.

The TSHR cryo-EM structure shows that Leu512^3.43^ in the TMD makes hydrophobic interactions with Ser508, Thr511, Thr513, Asp633 and Phe634 (superscripts refer to Ballesteros–Weinstein numbering; Ballesteros & Weinstein 1995). In addition, there are also interactions (hydrogen bond, polar and induced dipole) between Asp633^6.44^ and Asn674^7.49^. Residue Leu^3.43^ of the glycoprotein hormone receptor family is functionally important and is involved in a network of hydrophobic interactions in the inactive state. Furthermore, Asp^6.44^ which interacts with Asn^7.49^ in the inactive state of the GPHRs is Phe^6.44^ instead of Asp^6.44^ in most GPCRs (Tehan *et al.* 2014). Three naturally occurring TSHR Leu512^3.43^ polar mutants (Gln, Asn, Arg) that increase the constitutive activity of the receptor (Kosugi *et al.* 2000, Trülzsch *et al.* 2001, Nishihara *et al.* 2006) show reduced inter-helical packing. In addition, four naturally occurring TSHR Asp633^6.44^ mutants (Ala, Glu, His and Tyr) that increase the constitutive activity of the receptor (Porcellini *et al.* 1994, Russo *et al.* 1996, Parma *et al.* 1997) break or modify the interaction between Asp633^6.44^ and Asn674^7.49^. Mutational studies have shown that the interaction between Asp^6.44^ and Asn^7.49^ stabilises the inactive form of the TSHR TMD (Govaerts *et al.* 2001).

The complex of TSHR–K1-70™ Fab is seen as a monomer as is the case for the cryo-EM structure of LH/CGR in complex with hCG and G proteins (Duan *et al.* 2021). The LH/CGR complex is also described as being a rigid structure that would prevent receptor dimerisation (Duan *et al.* 2021) and this appears to be the case for TSHR complexed with K1-70™ Fab. However, there are reports that TSHR dimerisation and multimerisation occur (Urizar *et al.* 2005, Latif *et al.* 2015, Boutin *et al.* 2020).

A structural superimposition (Supplementary Fig. 5A, B and C) of the cryo-EM structure of the TSHR ECD with the crystal structure of the FSHR ECD (Fan & Hendrickson 2005) shows that they are similar to each other. Structural differences are observed in areas with amino acid insertions or deletions, in the N-terminal first two repeats, in the C-terminal cap and mostly in the flexible and unstructured long hinge loop.

A structural superimposition of the full-length TSHR and the full-length LH/CGR cryo-EM inactive structure (Supplementary Fig. 5D, E and F) determined byDuan *et al.* (2021) shows that the structures are similar. The functionally important linkers between the ECD and the TMD and the two long ECL1 and ECL2 loops are similar in both structures. The transmembrane helices show similar structures and relative positions except for TM6. There is a difference in the position of the extracellular end of TSHR TM6 which is displaced approximately 7 Å away from the centre of the helix bundle compared to the LH/CGR TM6 in the inactive structure. In contrast, there is no displacement of the intracellular end of TM6. The entire intracellular region of the TSHR TMD in our structure superimposes well with the equivalent region of the inactive LH/CGR structure. In both structures, access to the G-protein binding pocket is blocked by TM helix 6 which prevents the binding of G proteins and receptor activation. The inactive LH/CGR structure has the small molecule antagonist compound 26 bound within the TMD and this interacts with Ser586 and Met582 of the TM6 helix. In our structure of the TSHR bound to K1-70™, there is no antagonist in the allosteric binding pocket within the TMD for TM6 to interact with. It may be that the difference in the position of TM6 in the TSHR structure bound to K1-70™ and that seen in the LH/CGR is one of the reasons that the TSHR displays constitutive activity while LH/CGR does not (Van Sande *et al.* 1995).

Electron density is observed in the space in the TMD that is created by the displacement of TM6 of the TSHR in the TSHR–K1-70™ complex, but this does not fit with the structures of LMNG or CHS used in solubilisation and purification of the complex. Instead the electron density resembles that of a small phospholipid (Supplementary Fig. 3) which was most probably inside the receptor prior to solubilisation. Testing of potential phospholipid candidates for their ability to fit this electron density revealed inositol-1,4,5-triphosphate (IP_3_) as a good candidate which fits the electron density exactly. It is interesting to note that IP_3_ is a secondary signalling molecule produced by the activation of a Gq protein by GPCRs including the TSHR (Kleinau *et al.* 2010).

A structural superimposition of the full-length TSHR and the active LH/CGR structure (wild types LH/CGR in complex with hCG,Duan *et al.* 2021) shows an upward rotation of the ECD of the active LH/CGR by approximately 39^o^ around the hinge region compared to the TSHR ECD (calculated using Ser281 Cα–Glu61 Cα vector from the TSHR and Ser277 Cα–Tyr58 Cα vector from the LH/CGR) and an upward displacement of approximately 2 Å between TSHR Ser281 and LH/CGR Ser277. A comparison of the TMDs of both receptors shows that TM2, TM4 and TM8 superimpose on top of each other, whereas the positions of TM1 and TM3 show a small difference. In contrast, the position of the extracellular end of LH/CGR TM5 differs by 2 Å and the extracellular ends of TM6 and TM7 by 3 Å towards the centre of the helix bundle compared to the TSHR. Furthermore the position of the intracellular end of TM6 in the active structure of LH/CGR relative to the TSHR is approximately 12 Å away from the centre of the helix bundle typical of the difference between the active and inactive states of GPCRs (Choe *et al.* 2011). These differences in the two structures suggest that our structure of the TSHR bound to the blocking autoantibody K1-70™ is not that of a fully active GPCR.

Analysis of the cryo-EM structures of the inactive and the active LH/CGR complexes (Duan *et al.* 2021) shows a rotation in the ECD of the receptor occurs when hCG binds in a so-called ‘push’ movement. Furthermore, in the case of FSH binding to the FSHR (Jiang *et al.* 2012), a sulfonated tyrosine residue surrounded by a negatively charged patch in the receptor’s long hinge loop has been shown to bind to the hormone, pulling the ECD upwards. In the case of hCG binding to LH/CGR, a similar ‘pull’ mechanism is suggested to occur (Duan *et al.* 2021). The upward movement of the LH/CGR ECD resulting from these ‘push’ and ‘pull’ effects has been proposed to cause receptor activation (Duan *et al.* 2021). Also, ‘push’ and ‘pull’ mechanisms similar to that proposed for hCG binding to LH/CGR may be involved in the hormone activation of the TSHR and FSHR.

In contrast, Tyr385 of the TSHR which has been shown to be required for stimulation of the TSHR by TSH (is not required for stimulation of the TSHR by autoantibodies or mouse monoclonal stimulating antibodies (Kosogi *et al.* 1991, Costagliola *et al.* 2002). Consequently, binding of M22™ and other stimulating antibodies to the TSHR does not appear to cause a corresponding ‘pull’" effect via the hinge loop through Tyr385.

### Proposed mechanism of activation by M22

The crystal structure of TSHR260 in complex with the thyroid-stimulating human monoclonal TRAb M22™ shows that M22™ interacts with TSHR amino acids more C-terminal than those that interact with K1-70™ (Sanders *et al.* 2011). For example, TSHR Trp258 forms important interactions with the M22™ light chain, whereas Trp258 is not involved in K1-70™ binding.

**Figure 4:**
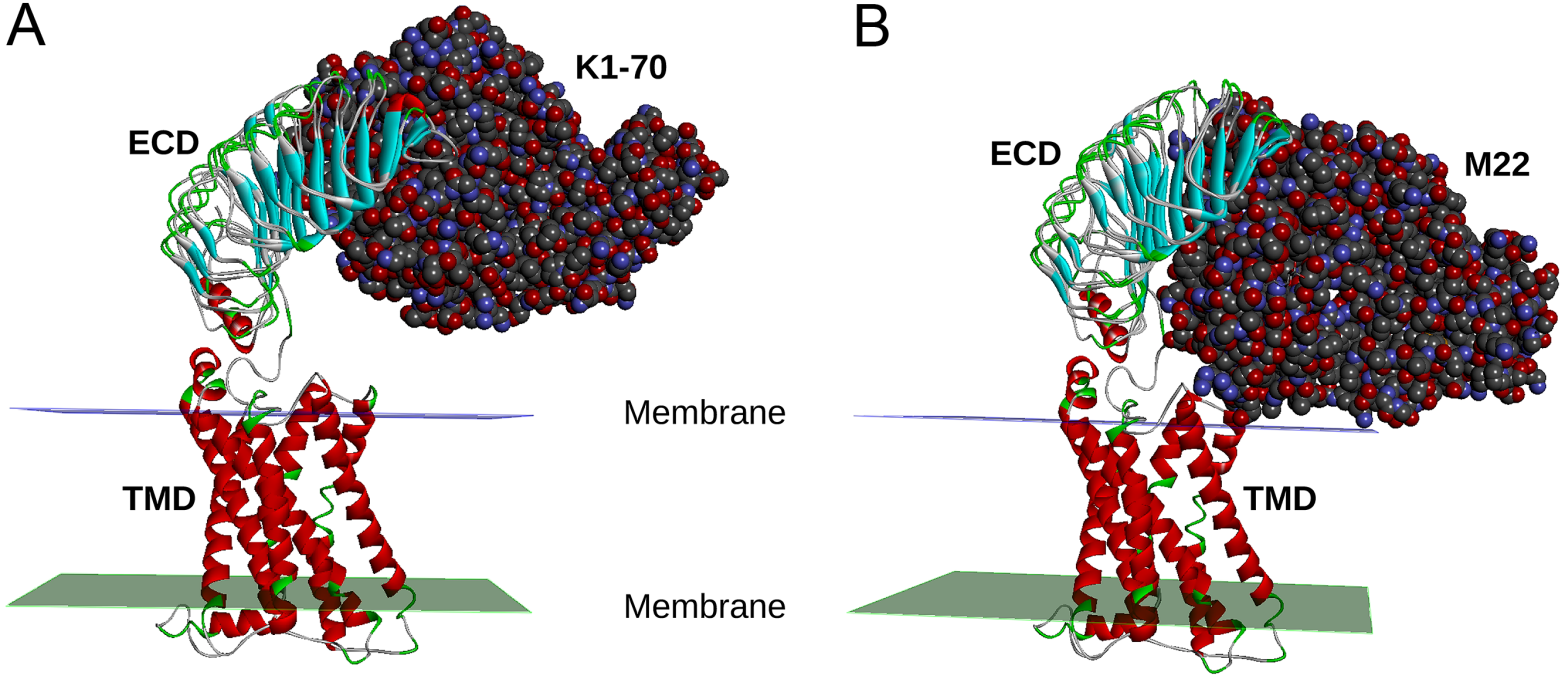
Proposed mechanism of TSHR activation by M22™. The cryo-EM structure of the TSHR bound to the blocking autoantibody K1-70™ was superimposed with the crystal structure of the TSHR leucine-rich domain (LRD) in complex with K1-70™ (A) and with the crystal structure of the TSHR LRD in complex with the stimulating monoclonal autoantibody M22™ (B). K1-70™ does not contact the membrane while the stimulating antibody M22™ is seen to clash with the membrane. This clash between M22™ and the membrane would inhibit M22™ binding to the TSHR unless the receptor’s ECD rotates upwards as part of the M22™ binding process. This rotation could have an important role in TSHR stimulation by M22™.

The implications of the differences in the TSHR LRD binding region of M22™ (more C-terminal) and K1-70™ (more N-terminal) can now be accessed using our cryo-EM full-length TSHR structure (Fig. 4). K1-70™ is well clear of the lipid bilayer in the TSHR–K1-70™ complex, whereas M22™ binding will result in a clash of the antibody light chain with the lipid bilayer unless the TSHR rotates upwards (i.e. away from the lipid bilayer) in the M22™ binding process. A similar ‘push’ process occurs when hCG binds to the LH/CG receptor (Duan *et al.* 2021) as mentioned earlier and stimulation of the TSHR by M22™ may also be caused by movements associated with such a ‘push’ process. This ‘push’ process would also explain the observations ofChazenbalk *et al.* (2002) who noted that thyroid-stimulating antibodies but not thyroid-blocking antibodies were partially sterically hindered when binding to the full-length TSHR compared to the TSHR ECD alone. One of their suggestions was that partial obstruction of the thyroid-stimulating autoantibody binding site may lead to a torsion effect on the TSHR ECD, on antibody binding, which may explain how thyroid-stimulating autoantibodies were able to activate the TSHR. Furthermore, Jiang *et al.* (2014) reported that their trimer model of full-length FSHR if applied to a TSHR trimer may explain the difference in the activities of M22™ (stimulating) and K1-70™ (blocking) autoantibodies, where M22™ but not K1-70™ would sterically clash with the TSHR hinge loop.

The cryo-EM structure of the full-length TSHR in complex with the powerful blocking type monoclonal autoantibody K1-70™ (Figs 3 and 4) indicates that when K1-70™ binds to the TSHR LRD it does not form interactions with other parts of the receptor. This confirms that the ability of K1-70™ to prevent activation of the TSHR by TSH or by stimulating autoantibodies is due to the direct binding of K1-70™ to the same region of the TSHR LRD so that binding of other ligands is blocked. This understanding of the mechanism of action of K1-70™ is helpful in developing applications for its use in controlling activation of the TSHR in Graves’ disease (Furmaniak *et al.* 2022), in Graves’ orbitopathy (Furmaniak *et al.* 2022) and in thyroid cancer (Ryder *et al.* 2021).

## Supplementary Material

Supplementary Material

## Declaration of interest

RSR Ltd is a developer and manufacturer of *in vitro* medical diagnostics including kits for measuring autoantibodies to the TSHR. The work was carried out while all authors were employees of RSR Ltd.

## Funding

The work was funded by RSR Ltd.

## References

[bib1] Bahn RS 2010 Graves’ ophthalmopathy. New England Journal of Medicine 362 726–738. (10.1056/NEJMra0905750) 20181974 PMC3902010

[bib2] Ballesteros JA Weinstein H 1995 Integrated methods for the construction of three dimensional models and computational probing of structure function relations in G protein-coupled receptors. Methods in Neurosciences 25 366–428. (10.1016/S1043-9471(0580049-7)

[bib3] BIOVIA Dassault Systèmes 2021 Discovery Studio 2021. San Diego, CA, USA: BIOVIA Dassault Systèmes.

[bib4] Birk HW Koepsell H 1987 Reaction of monoclonal antibodies with plasma membrane proteins after binding on nitrocellulose: renaturation of antigenic sites and reduction of non-specific antibody binding. Analytical Biochemistry 164 12–22. (10.1016/0003-2697(8790360-5) 2445218

[bib5] Boutin A Krieger CC Marcus-Samuels B Klubo-Gwiezdzinska J Neumann S Gershengorn MC 2020 TSH receptor homodimerization in regulation of cAMP production in human thyrocytes in vitro. Frontiers in Endocrinology 11 276. (10.3389/fendo.2020.00276) PMC7203478 32425890

[bib6] Brüser A Schulz A Rothemund S Ricken A Calebiro D Kleinau G Schöneberg T 2016 The activation mechanism of glycoprotein hormone receptors with implications in the cause and therapy of endocrine diseases. Journal of Biological Chemistry 291 508–520. (10.1074/jbc.M115.701102) 26582202 PMC4705372

[bib7] Chazenbalk GD Tanaka K Nagayama Y Kakinuma A Jaume JC McLachlan SM Rapoport B 1997 Evidence that the thyrotropin receptor ectodomain contains not one, but two, cleavage sites. Endocrinology 138 2893–2899. (10.1210/endo.138.7.5259) 9202233

[bib8] Chazenbalk GD Pichurin P Chen CR Latrofa F Johnstone AP McLachlan SM Rapoport B 2002 Thyroid-stimulating autoantibodies in Graves preferentially recognise free A subunit, not the thyrotropin holoreceptor. Journal of Clinical Investigation 110 209–217. (10.1172/JCI15745) 12122113 PMC151066

[bib9] Choe HW Kim YJ Park JH Morizumi T Pa EF Krauss N Hofmann KP Scheerer P Ernst OP 2011 Crystal structure of metarhodopsin II. Nature 471 651–655. (10.1038/nature09789) 21389988

[bib10] Costagliola S Panneels V Bonomi M Koch J Many MC Smits G Vassart G 2002 Tyrosine sulfation is required for agonist recognition by glycoprotein hormone receptors. EMBO Journal 21 504–513. (10.1093/emboj/21.4.504) 11847099 PMC125869

[bib11] Davies TF Ali MR Latif R 2014 Allosteric modulators hit the TSH receptor. Endocrinology 155 1–5. (10.1210/en.2013-2079) 24364583 PMC3868806

[bib12] Duan J Xu P Cheng X Mao C Croll T He X Shi J Luan X Yin W You E 2021 Structures of full-length glycoprotein hormone receptor signalling complexes. Nature 598 688–692. (10.1038/s41586-021-03924-2) 34552239

[bib13] Emsley P Lohkamp B Scott WG Cowtan K 2010 Features and development of coot. Acta Crystallographica: Section D, Biological Crystallography 66 486–501. (10.1107/S0907444910007493) 20383002 PMC2852313

[bib14] Evans M Sanders J Tagami T Sanders P Young S Roberts E Wilmot J Hu X Kabelis K Clark J 2010 Monoclonal autoantibodies to the TSH receptor, one with stimulating activity and one with blocking activity, obtained from the same blood sample. Clinical Endocrinology 73 404–412. (10.1111/j.1365-2265.2010.03831.x) 20550534

[bib15] Fan QR Hendrickson WA 2005 Structure of human follicle-stimulating hormone in complex with its receptor. Nature 433 269–277. (10.1038/nature03206) 15662415 PMC5514322

[bib16] Furmaniak J Sanders J Núñez Miguel R Rees Smith B 2015 Mechanisms of action of TSHR autoantibodies. Hormone and Metabolic Research 47 735–752. (10.1055/s-0035-1559648) 26361260

[bib17] Furmaniak J Sanders J Clark J Wilmot J Sanders P Li Y Rees Smith B 2019 Preclinical studies on the toxicology, pharmacokinetics and safety of K1-70™ a human monoclonal autoantibody to the TSH receptor with TSH antagonist activity. Auto-Immunity Highlights 10 11. (10.1186/s13317-019-0121-9) PMC7065368 32257067

[bib18] Furmaniak J Sanders J Sanders P Li Y Rees Smith B 2022 TSH receptor specific monoclonal autoantibody K1-70™ targeting of the TSH receptor in subjects with Graves’ disease and Graves’ orbitopathy – results from a phase I clinical trial. Clinical Endocrinology 96 878–887. (10.1111/cen.14681) 35088429 PMC9305464

[bib19] Govaerts C Lefort A Costagliola S Wodak SJ Ballesteros JA Van Sande J Pardo L Vassart G 2001 A conserved ASN in transmembrane helix 7 is an on/off switch in the activation of the thyrotropin receptor. Journal of Biological Chemistry 276 22991–22999. (10.1074/jbc.M102244200) 11312274

[bib20] Jiang X Liu H Chen X Chen PH Fischer D Sriraman V Yu HN Arkinstall S He X 2012 Structure of follicle-stimulating hormone in complex with the entire ectodomain of its receptor. PNAS 109 12491–12496. (10.1073/pnas.1206643109) 22802634 PMC3411987

[bib21] Jiang X Fischer D Chen X McKenna SD Liu H Sriraman V Yu HN Goutopoulos A Arkinstall S He X 2014 Evidence for follicle-stimulating hormone receptor as a functional trimer. Journal of Biological Chemistry 289 14273–14282. (10.1074/jbc.M114.549592) 24692546 PMC4022893

[bib22] Katritch V Cherezov V Stevens RC 2012 Diversity and modularity of G protein-coupled receptor structures. Trends in Pharmacological Sciences 33 17–27. (10.1016/j.tips.2011.09.003) 22032986 PMC3259144

[bib23] Kleinau G Claus M Jaeschke H Mueller S Neumann S Paschke R Krause G 2007 Contacts between extracellular loop two and transmembrane helix six determine basal activity of the thyroid-stimulating hormone receptor. Journal of Biological Chemistry 282 518–525. (10.1074/jbc.M606176200) 17079233

[bib24] Kleinau G Jaeschke H Worth CL Mueller S Gonzalez J Paschke R Krause G 2010 Principles and determinants of G-protein coupling by the rhodopsin-like thyrotropin receptor. PLoS ONE 5 e9745. (10.1371/journal.pone.0009745) PMC2841179 20305779

[bib25] Kleinau G Worth CL Kreuchwig A Biebermann H Marcinkowski P Scheerer P Krause G 2017 Structural-functional features of the thyrotropin receptor: a class A G-protein-coupled receptor at work. Frontiers in Endocrinology 8 86. (10.3389/fendo.2017.00086) PMC5401882 28484426

[bib26] Kosogi S Ban T Akamizu T Kohn LD 1991 Further characterisation of a high affinity thyrotropin binding site on the rat thyrotropin receptor which is an epitope for blocking antibodies from idiopathic myxedema patients but not thyroid stimulating antibodies from Graves’ patients. Biochemical and Biophysical Research Communications 180 1118–1124. (10.1016/S0006-291X(0581182-9) 1719963

[bib27] Kosugi S Hai N Okamoto H Sugawa H Mori T 2000 A novel activating mutation in the thyrotropin receptor gene in an autonomously functioning thyroid nodule developed by a Japanese patient. European Journal of Endocrinology 143 471–477. (10.1530/eje.0.1430471) 11022192

[bib28] Krause G Eckstein A Schülein R 2020 Modulating TSH receptor signaling for therapeutic benefit. European Thyroid Journal 9 66–77. (10.1159/000511871) 33511087 PMC7802447

[bib29] Laemmli UK 1970 Cleavage of structural proteins during the assembly of the head of bacteriophage T4. Nature 227 680–685. (10.1038/227680a0) 5432063

[bib30] Latif R Ali MR Mezei M Davies TF 2015 Transmembrane domains of attraction on the TSH receptor. Endocrinology 156 488–498. (10.1210/en.2014-1509) 25406938 PMC4298320

[bib31] Miller-Gallacher J Sanders P Young S Sullivan A Baker S Reddington SC Clue M Kabelis K Clark J Wilmot J 2019 Crystal structure of a ligand-free stable TSH receptor leucine-rich repeat domain. Journal of Molecular Endocrinology 62 117–128. (10.1530/JME-18-0213) 30689545

[bib32] Morshed SA Latif R Davies TF 2012 Delineating the autoimmune mechanisms in Graves’ disease. Immunologic Research 54 191–203. (10.1007/s12026-012-8312-8) 22434518 PMC4504182

[bib33] Nishihara E Fukata S Hishinuma A Kudo T Ohye H Ito M Kubota S Amino N Kuma K Miyauchi A 2006 Sporadic congenital hyperthyroidism due to a germline mutation in the thyrotropin receptor gene (Leu 512 Gln) in a Japanese patient. Endocrine Journal 53 735–740. (10.1507/endocrj.k06-090) 16960398

[bib34] Núñez Miguel R Sanders J Sanders P Young S Clark J Kabelis K Wilmot J Evans M Roberts E Hu X 2012 Similarities and differences in interactions of thyroid stimulating and blocking autoantibodies with the TSH receptor. Journal of Molecular Endocrinology 49 137–151. (10.1530/JME-12-0040) 22829655

[bib35] Núñez Miguel R Sanders J Furmaniak J Rees Smith BR 2017a Structure and activation of the TSH receptor transmembrane domain. Auto-Immunity Highlights 8 2. (10.1007/s13317-016-0090-1) PMC5136658 27921237

[bib36] Núñez Miguel R Sanders J Furmaniak J Rees Smith B 2017b Glycosylation pattern analysis of glycoprotein hormones and their receptors. Journal of Molecular Endocrinology 58 25–41. (10.1530/JME-16-0169) 27875255

[bib37] Oda Y Sanders J Roberts S Maruyama M Kato R Perez M Petersen VB Wedlock N Furmaniak J Rees Smith B 1998 Binding characteristics of antibodies to the TSH receptor. Journal of Molecular Endocrinology 20 233–244. (10.1677/jme.0.0200233) 9584838

[bib38] Oda Y Sanders J Evans M Kiddie A Munkley A James C Richards T Wills J Furmaniak J Smith BR 2000 Epitope analysis of the human thyrotropin (TSH) receptor using monoclonal antibodies. Thyroid 10 1051–1059. (10.1089/thy.2000.10.1051) 11201849

[bib39] Parma J Van Sande J Swillens S Tonacchera M Dumont J Vassart G 1995 Somatic mutations causing constitutive activity of the thyrotropin receptor are the major cause of hyperfunctioning thyroid adenomas: identification of additional mutations activating both the cyclic adenosine 3’,5’-monophosphate and inositol phosphate-Ca^2+^ cascades. Molecular Endocrinology 9 725–733. (10.1210/mend.9.6.8592518) 8592518

[bib40] Parma J Duprez L Van Sande J Hermans J Rocmans P Van Vliet G Costagliola S Rodien P Dumont JE Vassart G 1997 Diversity and prevalence of somatic mutations in the thyrotropin receptor and Gs alpha genes as a cause of toxic thyroid adenomas. Journal of Clinical Endocrinology and Metabolism 82 2695–2701. (10.1210/jcem.82.8.4144) 9253356

[bib41] Porcellini A Ciullo I Laviola L Amabile G Fenzi G Avvedimento VE 1994 Novel mutations of thyrotropin receptor gene in thyroid hyperfunctioning adenomas. Rapid identification by fine needle aspiration biopsy. Journal of Clinical Endocrinology and Metabolism 79 657–661. (10.1210/jcem.79.2.8045989) 8045989

[bib42] Punjani A Rubinstein JL Fleet DJ Brubaker MA 2017 cryoSPARC: algorithms for rapid unsupervised cryo-EM structure determination. Nature Methods 14 290–296. (10.1038/nmeth.4169) 28165473

[bib43] Rapoport B Chazenbalk GD Jaume JC McLachlan SM 1998 The thyrotropin (TSH) receptor: interaction with TSH and autoantibodies. Endocrine Reviews 19 673–716. (10.1210/edrv.19.6.0352) 9861544

[bib44] Rees Smith B McLachlan SM Furmaniak J 1988 Autoantibodies to the thyrotropin receptor. Endocrine Reviews 9 106–121. (10.1210/edrv-9-1-106) 3286231

[bib45] Russo D Arturi F Suarez HG Schlumberger M Du Villard JA Crocetti U Filetti S 1996 Thyrotropin receptor gene alterations in thyroid hyperfunctioning adenomas. Journal of Clinical Endocrinology and Metabolism 81 1548–1551. (10.1210/jcem.81.4.8636365) 8636365

[bib46] Ryder M Wentworth M Algeciras-Schimnich A Morris JC Garrity J Sanders J Young S Sanders P Furmaniak J Rees Smith B 2021 Blocking the thyrotropin receptor with K1-70 in a patient with follicular thyroid cancer, Graves’ disease, and Graves’ ophthalmopathy. Thyroid 31 1597–1602. (10.1089/thy.2021.0053) 34114495

[bib47] Sali A Blundell TL 1993 Comparative protein modelling by satisfaction of spatial restraints. Journal of Molecular Biology 234 779–815. (10.1006/jmbi.1993.1626) 8254673

[bib48] Sanders J Evans M Premawardhana L Depraetere H Jeffreys J Richards T Furmaniak J Rees Smith BR 2003 Human monoclonal thyroid stimulating autoantibody. Lancet 362 126–128. (10.1016/S0140-6736(0313866-4) 12867115

[bib49] Sanders J Chirgadze DY Sanders P Baker S Sullivan A Bhardwaja A Bolton J Reeve M Nakatake N Evans M 2007 Crystal structure of the TSH receptor in complex with a thyroid-stimulating autoantibody. Thyroid 17 395–410. (10.1089/thy.2007.0034) 17542669

[bib50] Sanders P Young S Sanders J Kabelis K Baker S Sullivan A Evans M Clark J Wilmot J Hu X 2011 Crystal structure of the TSH receptor bound to a blocking type TSHR autoantibody. Journal of Molecular Endocrinology 46 81–99. (10.1530/JME-10-0127) 21247981

[bib51] Schaarschmidt J Nagel MBM Huth S Jaeschke H Moretti R Hintze V Von Bergen M Kalkhof S Meiler J Paschke R 2016 Rearrangement of the extracellular domain/extracellular loop1 interface is critical for thyrotropin receptor activation. Journal of Biological Chemistry 291 14095–14108. (10.1074/jbc.M115.709659) 27129207 PMC4933169

[bib52] Tegunov D Cramer P 2019 Real-time cryo-electron microscopy data preprocessing with warp. Nature Methods 16 1146–1152. (10.1038/s41592-019-0580-y) 31591575 PMC6858868

[bib53] Tehan BG Bortolato A Blaney FE Weir MP Mason JS 2014 Unifying family A GPCR theories of activation. Pharmacology and Therapeutics 143 51–60. (10.1016/j.pharmthera.2014.02.004) 24561131

[bib54] Trülzsch B Krohn K Wonerow P Chey S Holzapfel HP Ackermann F Führer D Paschke R 2001 Detection of thyroid-stimulating hormone receptor and Gsalpha mutations: in 75 toxic thyroid nodules by denaturing gradient gel electrophoresis. Journal of Molecular Medicine 78 684–691. (10.1007/s001090000170) 11434721

[bib55] Tunyasuvunakool K Adler J Wu Z Green T Zielinski M Žídek A Bridgland A Cowie A Meyer C Laydon A 2021 Highly accurate protein structure prediction for the human proteome. Nature 596 590–596. (10.1038/s41586-021-03828-1) 34293799 PMC8387240

[bib56] Urizar E Montanelli L Loy T Bonomi M Swillens S Gales C Bouvier M Smits G Vassart G Costagliola S 2005 Glycoprotein hormone receptors: link between receptor homodimerization and negative cooperativity. EMBO Journal 24 1954–1964. (10.1038/sj.emboj.7600686) 15889138 PMC1142614

[bib57] Van Sande J Parma J Tonacchera M Swillens S Dumont J Vassart G 1995 Somatic and germline mutations of the TSH receptor gene in thyroid diseases. Journal of Clinical Endocrinology and Metabolism 80 2577–2585. (10.1210/jcem.80.9.7673398) 7673398

[bib58] Webb B Sali A 2016 Comparative protein structure modeling using modeller. Current Protocols in Bioinformatics 54 5.6.1–5.6.37. (10.1002/cpbi.3) PMC5031415 27322406

[bib59] Zhang H Qiao A Yang D Yang L Dai A de Graaf C Reedtz-Runge S Dharmarajan V Zhang H Han GW 2017 Structure of the full-length glucagon class B G-protein-coupled receptor. Nature 546 259–264. (10.1038/nature22363) 28514451 PMC5492955

[bib60] Zhang H Qiao A Yang L Van Eps N Frederiksen KS Yang D Dai A Cai X Zhang H Yi C 2018 Structure of the glucagon receptor in complex with a glucagon analogue. Nature 553 106–110. (10.1038/nature25153) 29300013

