# Supplementary material for: Structure of full-length TSH receptor in complex with antibody K1-70™
Source: J Mol Endocrinol. 2022 Sep 6;70(1):e220120. doi: 10.1530/JME-22-0120 (PMC9782461; doi:10.1530/JME-22-0120)

Supplementary Table 1 Cryo-EM data collection, refinement and validation statistics

**Data collection and processing**	
Detector	Gatan K3
Magnification	130k
Voltage (kV)	300
Flux on detector (e/pix/sec)	15.11
Electron exposure on sample (e–/Å^2)	47.63
Target defocus range (μm)	1.0-2.4
Calibrated pixel size (Å)	0.652
Symmetry imposed	C1
Extraction box size (pixels)	400
Initial particle images (no.)	1616076
Final particle images (no.)	114410
**Refinement**	
Map resolution at FSC=0.143 (Å)	3.32
Model composition	
Non-hydrogen atoms	8220
Protein residues	1015
Nucleotides	0
B factor (Å^2)	
Protein	120.58
Ligand	-
R.m.s deviations	
Bond lengths (Å)	0.007
Bond angles (°)	1.156
Validation	
Molprobity score	3.63
Clashscore	28.31
Poor rotamers (%)	33.74
Ramachandran plot	
Favored (%)	90.64
Allowed (%)	98.92
Disallowed (%)	1.08

Supplementary Table 2 RMSD of residues 30 – 257 (present in all structures) between the TSHR LRDs from: the TSHR-K1-70 cryo-EM structure (TSHR-C), the TSHR LRD-K1-70 crystal structure (TSHR-K), the TSHR LRD-M22 crystal structure (TSHR-M) and the ligand-free stable TSHR-LRD (TSHR-B).

		RMSD of Cα atoms (Å)			
		TSHR-C	TSHR-K	TSHR-M	TSHR-B
RMSD of backbone atoms (Å)	TSHR-C	-	0.57	0.67	0.62
	TSHR-K	0.65	-	0.51	0.59
	TSHR-M	0.75	0.55	-	0.70
	TSHR-B	0.71	0.60	0.74	-

Supplementary Figure 1 Cryo-EM images and electron density map of the TSHR—K1-70 ^{TM} complex.

A Cryo-EM imaging on Titan Krios showing particles of the complex. 8,219 movies were collected and WARP identified 1,600,000 particles

B Data analysis in CryoSPARC showing 2D classification. Average images of the 2D projections of the particles picked by WARP.

C, D TSHR—K1-70^{TM} complex map at 3.32Å overall resolution showing the detergent belt around the transmembrane domain (TMD). Lower contour levels of the overall map show the transmembrane helices.

E The K1-70^{TM} Fab and the TSHR extracellular domain (ECD) were refined independently of the TMD to achieve a local resolution of 3.26Å.

F The locally refined map of the ECD was combined with the overall map to produce the final composite map

G Cryo-EM density map and model of the TSHR—K1-70^{TM} complex. The regions of the cryo-EM density map are coloured by local resolutions from 2.92Å (red) to 7.3Å (blue). The resolution of the overall density map is 3.32Å.

Supplementary Figure 1A

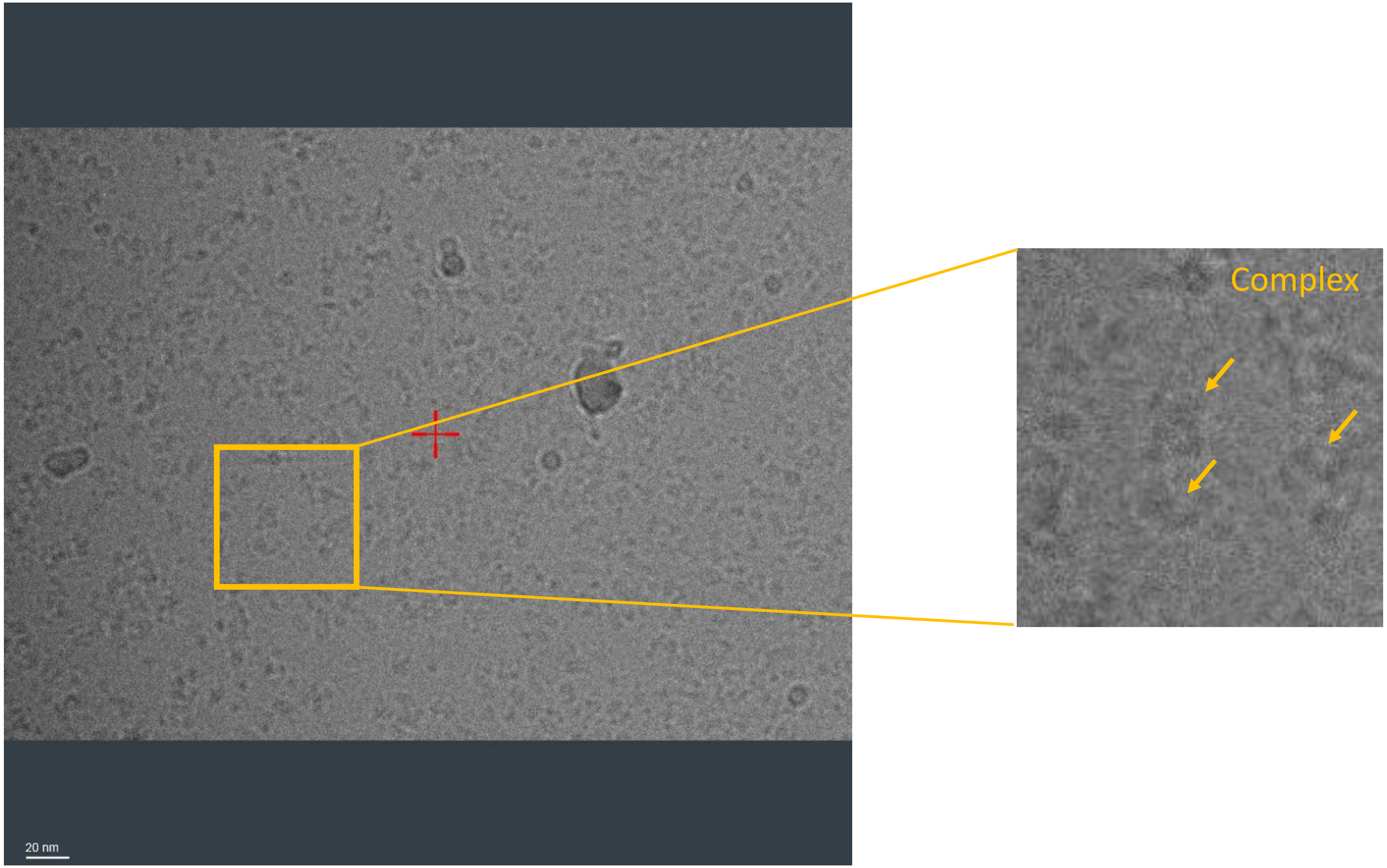

Supplementary Figure 1B

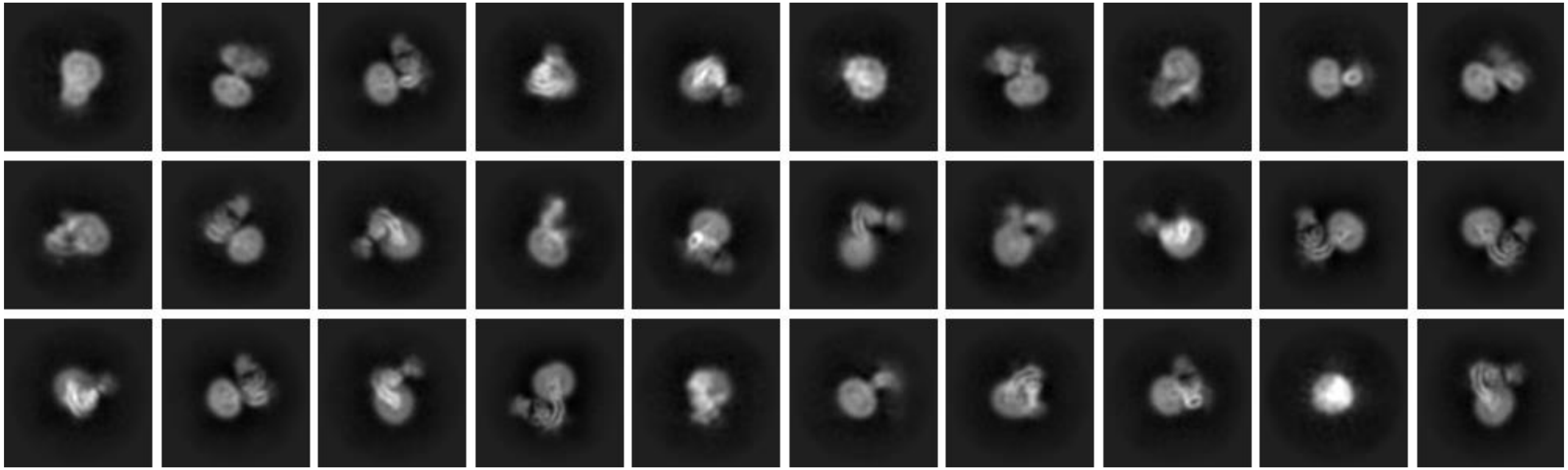

Supplementary Figure 1C

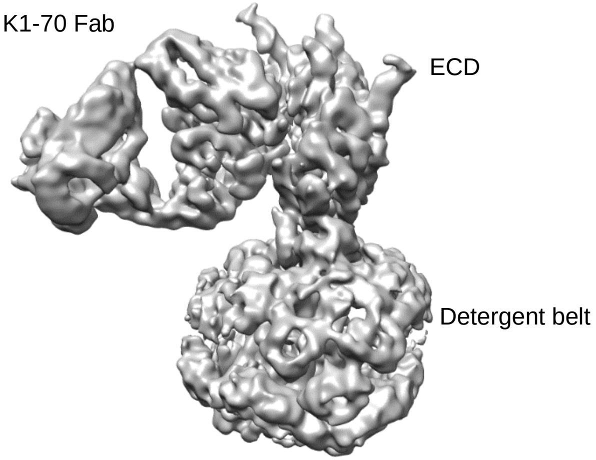

Final map at 3.32Å overall resolution. Detergent belt visible around transmembrane region

Supplementary Figure 1D

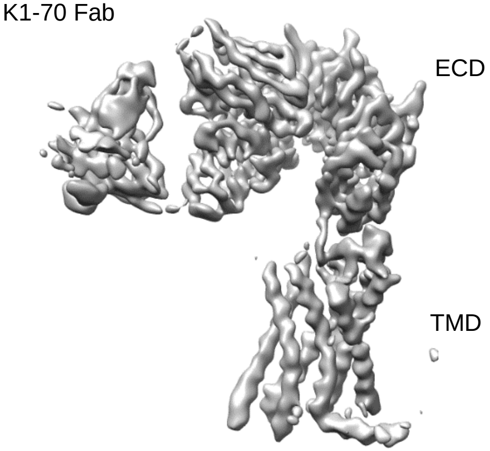

Transmembrane helices distinguishable at lower contour levels

Supplementary Figure 1E

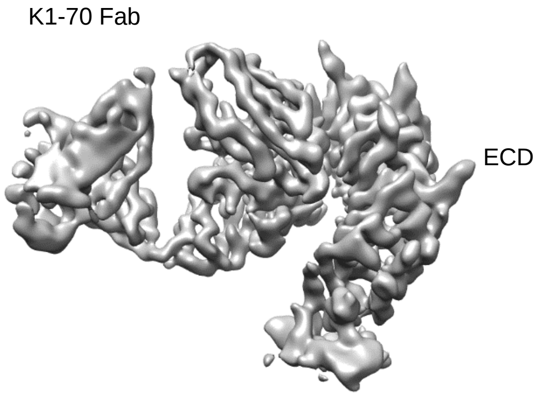

Non-membrane region refined independently of membrane region to achieve local resolution of 3.26Å

Supplementary Figure 1F

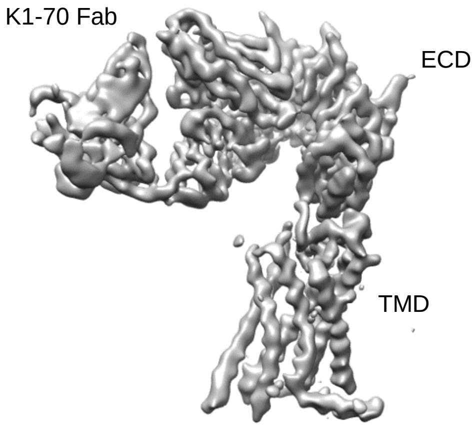

Locally refined map combined with overall map to produce final composite map

Supplementary Figure 1G

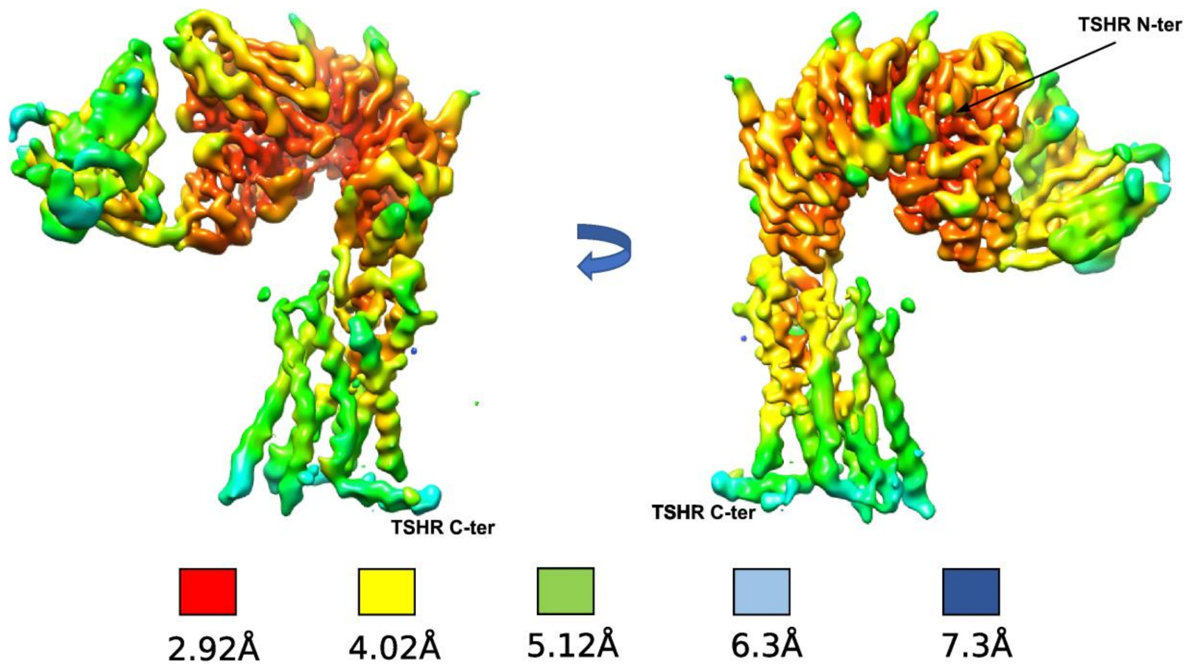

Supplementary Figure 2 Structural features of the full length TSHR

A The TSHR extracellular domain (ECD) consisting of the LRD and HR in 3 views rotated $90^{o.}$ The structure is in cartoon representation with the disulphide bonds and glycosylation sites in ball and stick representation with oxygen in red, nitrogen in blue and sulfur in yellow. The glycans are shown in purple.

B The TMD in 3 different orientations showing the transmembrane domain helices, extracellular and intracellular loops. The structure is in cartoon representation with the disulphide bonds in ball and stick representation as above.

Supplementary Figure 2A

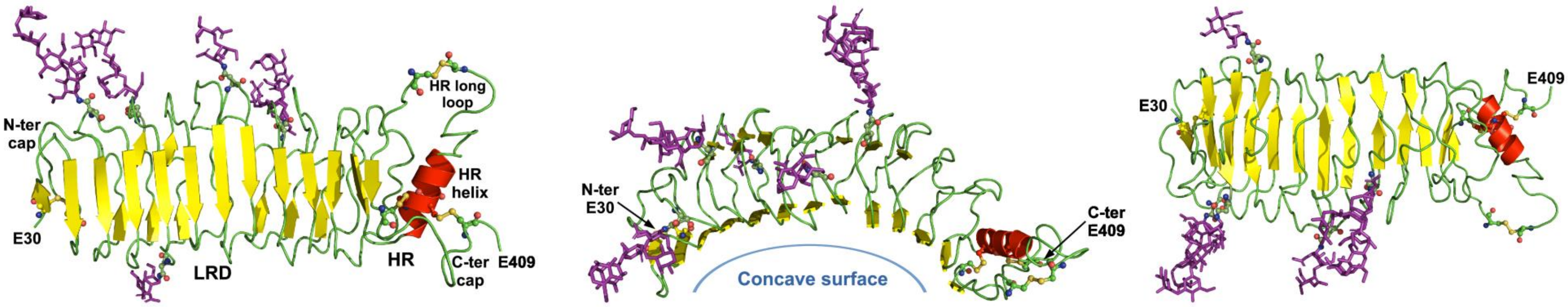

Supplementary Figure 2B

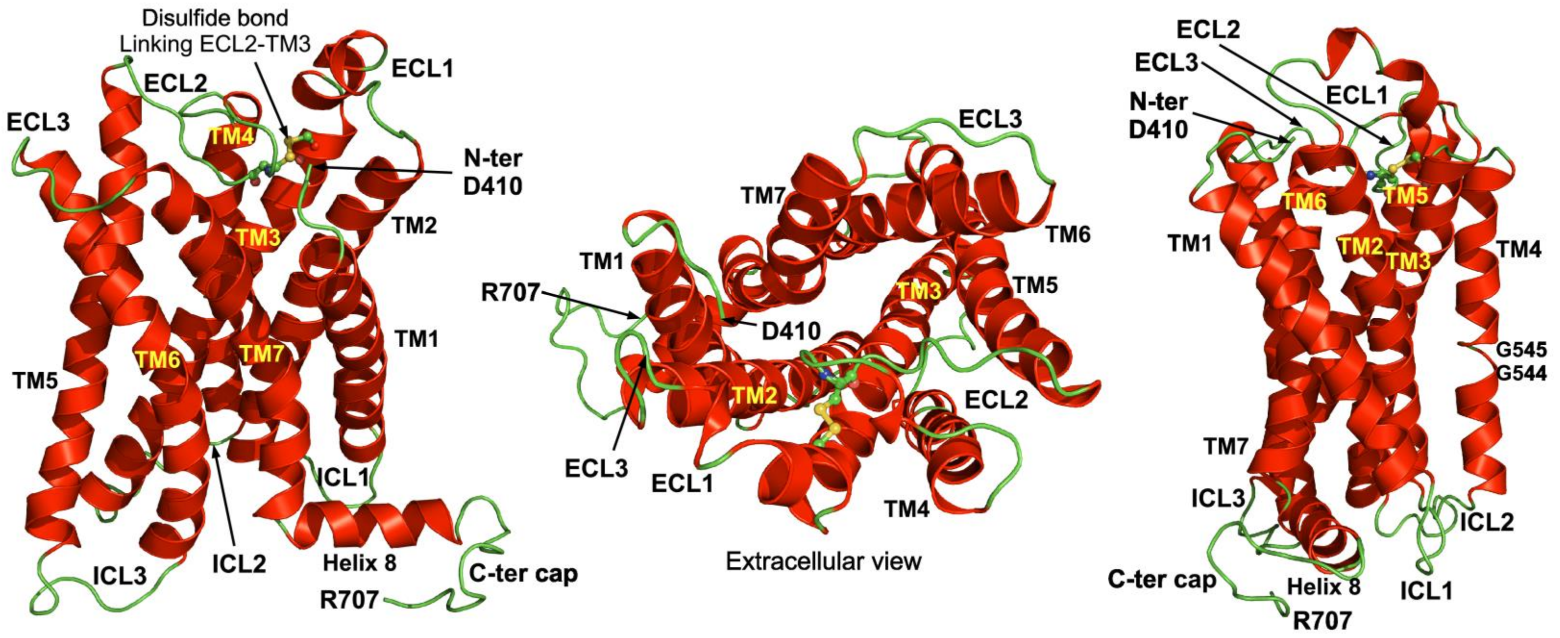

Supplementary Figure 3 Electron density map of the TMD showing density of a small phospholipid within the transmembrane domain pocket. Inositol-1,4,5-triphosphate (IP_3) is a good fit for the electron density.

Supplementary Figure 3

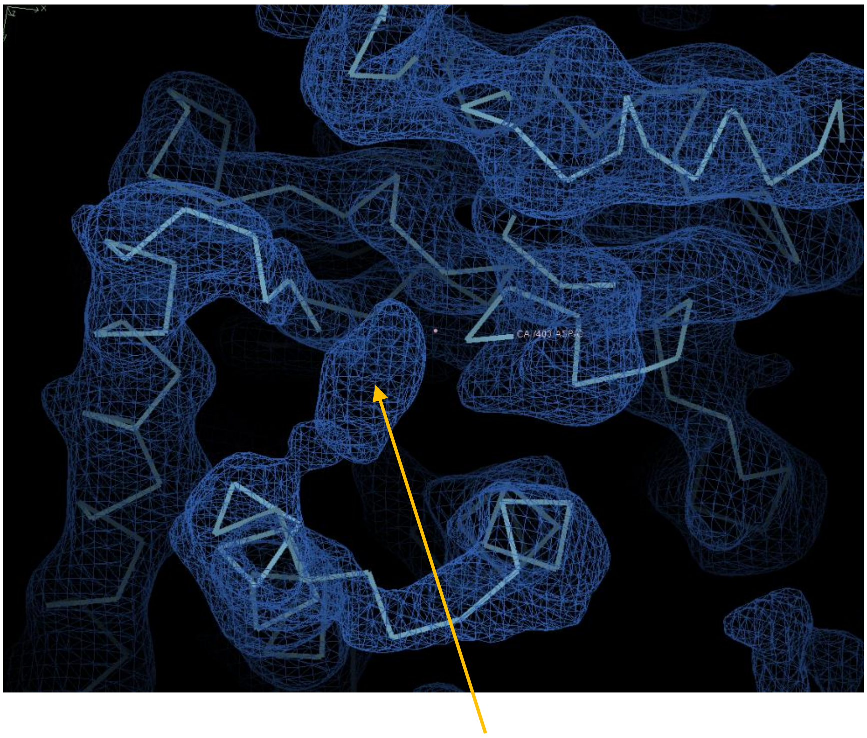

Electron density within transmembrane pocket

Supplementary Figure 4 Structural features of the full length TSHR in complex with the blocking monoclonal autoantibody K1-70^{TM}.

The TSHR—K1-70^{TM} Fab structure in 3 views rotated approximately 90°. The structure is in cartoon representation with the glycans shown in purple sticks. The K1-70^{TM} heavy chain is shown in blue, the K1-70^{TM} light chain in light green and the TSHR in green. The TSHR N terminus, extracellular domain (ECD), the transmembrane domain (TMD) and the intra cellular C terminus are marked.

Supplementary Figure 4

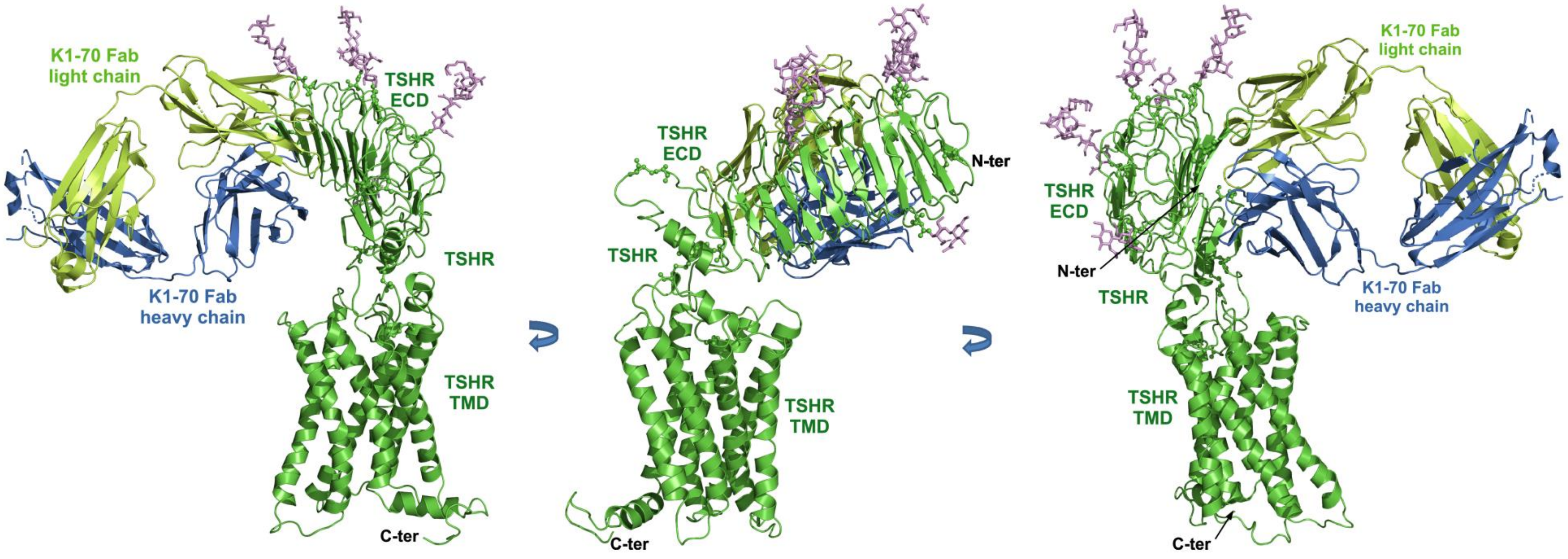

Supplementary Figure 5 Superimposition of the TSHR with the FSHR and LH/CGR

A-C Superimposition of the TSHR and the FSHR extracellular domains. The structures are in cartoon representation with the disulphide bonds in ball and stick representation with oxygen in red, nitrogen in blue and sulphur yellow. The TSHR ECD is shown in green and the FSHR in blue.

A shows the structure of the whole extracellular domain excluding part of the long hinge loop region which is not visible in the structures

B shows the differences at the N-terminus of both receptors

C shows the differences at the C-terminus of both receptors

D-F Superimposition of the cryo-EM structures of the full length TSHR from the K1-70™ complex with the full length inactive LH/CGR structure. The structures are in cartoon representation with the disulphide bonds in ball and stick representation with oxygen in red, nitrogen in blue and sulphur yellow. The TSHR is shown in green and the LH/CGR in orange.

D shows the structure of the full length receptors excluding part of the long hinge loop region which is not visible in the structures

E shows the extracellular loop 1 (ECL1) of the transmembrane domain (TMD) of both receptors

F the extracellular view of the TMD showing the displacement of TMD helix 6 in the TSHR structure compared to the inactive LH/CGR structure.

Supplementary Figure 5A

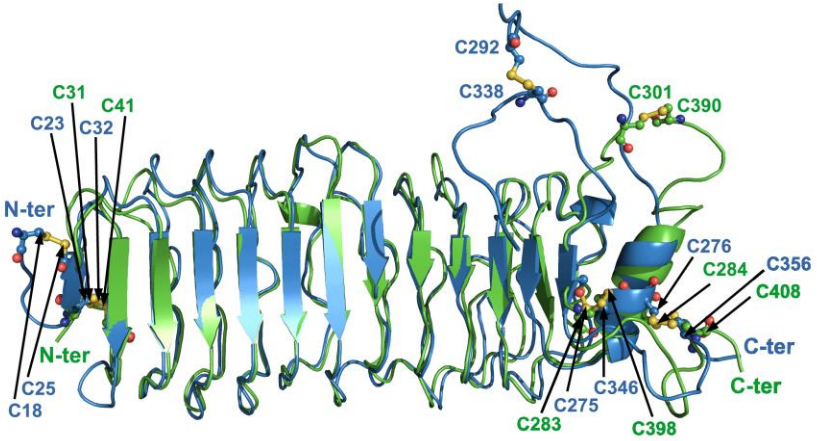

Supplementary Figure 5B

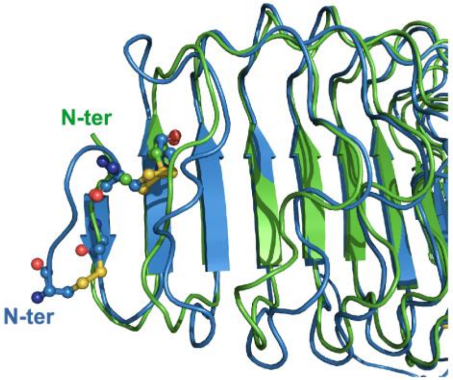

Supplementary Figure 5C

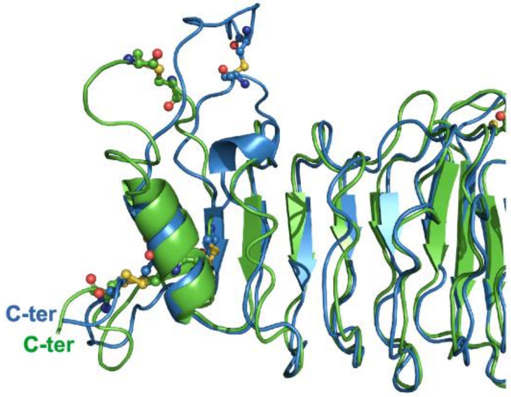

Supplementary Figure 5D

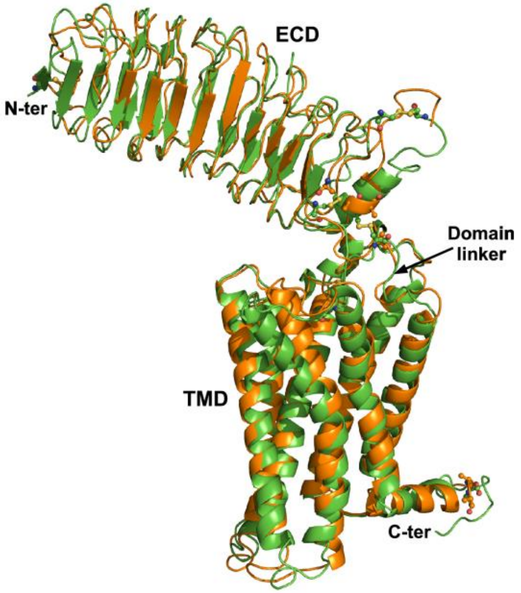

Supplementary Figure 5E

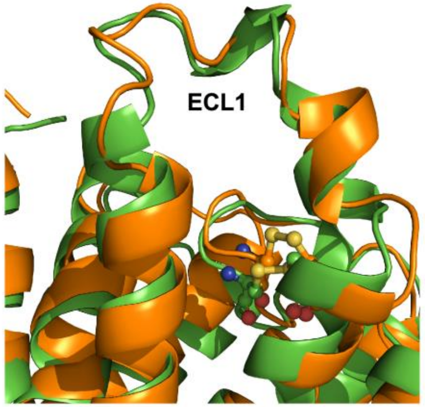

Supplementary Figure 5F

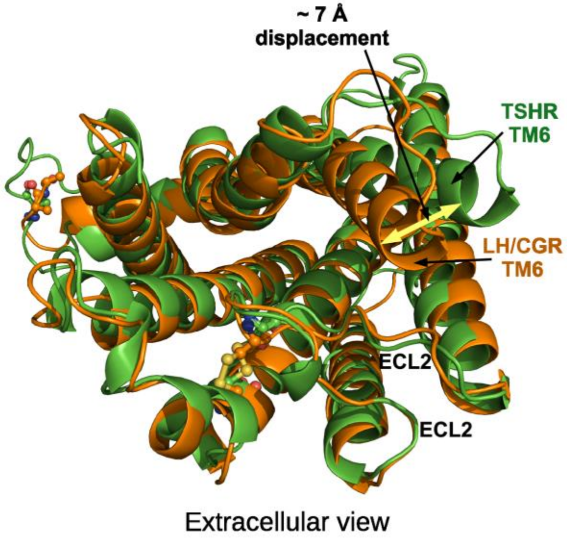

Supplement: Supplementary Material [file supplementary_material.pdf]
